# Responses of soil carbon storage to tending thinning and rapid prediction based on NIRS-BPNN in larch-birch natural secondary forests

**DOI:** 10.3389/fpls.2026.1926304

**Published:** 2026-08-26

**Authors:** Yongbin Meng, Yuanyuan Zhang, Xiangqian Zhang, Wenting Jiang, Yuanyuan Yang, Chunmei Chen

**Affiliations:** 1 School of Life Science, Yan’an University, Yan’an, China; 2 Key Laboratory of Applied Ecology of Loess Plateau, Shaanxi Province Higher Education Institutions, Yan‘an University, Yan’an, China

**Keywords:** BPNN, natural secondary forests, near-infrared spectroscopy, soil carbon storage, tending thinning

## Abstract

**Introduction:**

Heilongjiang Province ranks third in China in forest area, with over half of its forests being natural secondary forests. The province has fertile soil and plays a crucial role in China's forest ecosystem. Nevertheless, historical indiscriminate logging has resulted in degraded forest conditions, reduced productivity, and compromised carbon sequestration capacity.

**Methods:**

This research focused on the larch-birch natural secondary forest in the Greater Khingan Mountains, examining the soil twelve years after thinning. Six thinning treatments (0%, 16.7%, 25.5%, 34.4%, 49.6%, 59.9%) were compared to assess soil physical and chemical properties, soil carbon storage, and to elucidate the associations between thinning intensity and soil carbon storage dynamics. A soil organic carbon (SOC) content prediction model was developed using near-infrared spectroscopy and a Back Propagation neural network (BPNN) .

**Results:**

The findings indicate that thinning intensity significantly alters soil carbon storage by affecting key parameters such as soil moisture and organic carbon content. Specifically, a 34.4% thinning intensity significantly enhanced soil moisture content (56.67% vs. 33.02% in control, *p* < 0.05) and SOC content (66.17 g/kg vs. 32.03 g/kg in control, *p* < 0.05), leading to a substantial increase in total soil carbon storage (83.80 t/ha vs. 43.99 t/ha in control, *p* < 0.05). The NIRS-BPNN model achieved a prediction set correlation coefficient of *Rp* = 0.9293 (*R^2^p* = 0.8635, RMSEP = 0.0064, RPD = 2.8), demonstrating good predictive performance.

**Discussion:**

This research elucidates the responses of soil carbon storage to tending and thinning, providing a scientific foundation for the management of secondary forests. The NIRS-BPNN model offers a novel, rapid, and non-destructive approach for the large-scale assessment of SOC content in secondary forests, holding considerable theoretical and practical significance for forest carbon monitoring and climate change mitigation.

## 1 Introduction

Since the Industrial Revolution, the persistent increase in atmospheric CO_2_ concentrations has resulted in global warming, glacial melting, frequent wildfires, and extreme weather events, thereby initiating a series of positive feedback loops. As the largest carbon reservoir in terrestrial ecosystems, forests are crucial to the carbon cycle and the mitigation of climate change. In this context, forest soil serves as a significant carbon storage medium, and the dynamic fluctuations in its carbon storage directly influence the global carbon balance (Liao et al., 2024).

Secondary forests play a crucial role in the terrestrial carbon sink, yet they often face challenges like such as low productivity, limited carbon sequestration potential, and poor soil fertility, impeding sustainable forest development (Shi et al., 2024; Wang et al., 2024, 2025). Human interventions like tending and thinning can enhance habitat quality, stimulate tree growth, and foster regeneration, making them key considerations in worldwide efforts toward sustainable forest management and climate change mitigation (Cantarello et al., 2024; Ge and Lin, 2024; Lee et al., 2024).

Thinning enhances productivity and ecological functions by regulating stand structure; however, its effects on soil carbon storage remain contentious. Thinning can increase understory light and promote vegetation growth, thereby enhancing organic carbon input (Wang et al., 2024; Liang et al., 2025). Conversely, it may influence carbon fixation and release by altering microbial communities and soil physicochemical properties (Zhang et al., 2023; Shi et al., 2024; Xu et al., 2025). Thus, understanding the mechanisms by which thinning affects soil carbon storage is essential for optimizing forest management and improving carbon sink functions. International experiences, such as those from Russia and Japan, demonstrate that scientifically informed thinning can enhance forest carbon sinks, although the optimal intensity varies according to stand types (Meena et al., 2025; Qubaja et al., 2025). Existing studies suggest that high-intensity thinning (45%) may increase soil carbon storage (Wang et al., 2024), while moderate thinning could result in a reduction of surface carbon (Li et al., 2025; Wang et al., 2025b). Meta-analyses indicate that the thinning effect may not be significant (Zhang et al., 2023; Lee et al., 2024). These conflicting findings underscore the necessity for mechanistic studies, particularly concerning the impact of thinning on SOC components, including both physical and chemical forms, which remain poorly understood (Zhang et al., 2024b; De Quesada et al., 2025).

A global meta-analysis by Zhou et al. (2020) provides a critical microbial perspective. This study synthesized global data on the effects of thinning on soil microbial community structure and extracellular enzyme activities, revealing that thinning significantly alters microbial community composition, particularly by reducing the fungal-to-bacterial (F/B) ratio. This shift is crucial because fungi-dominated decomposition pathways are generally associated with the formation of more stable SOC. By altering the understory microenvironment (e.g., light, temperature, and moisture), thinning indirectly affects microbial substrate utilization efficiency and community functions, thereby regulating SOC decomposition and stabilization processes. These findings explain, from a microbial mechanistic perspective, why thinning effects vary widely across different ecosystems. Mäkipää et al. (2023) systematically evaluated the effects of various forest management practices—including thinning—on soil carbon sequestration and greenhouse gas fluxes in temperate and boreal forests. They pointed out that the net effect of thinning on soil carbon stocks depends on a complex trade-off: on the one hand, thinning reduces aboveground biomass, potentially causing short-term carbon losses; on the other hand, it promotes understory vegetation growth, increases litter input, and enhances root exudates, thereby augmenting soil carbon inputs. More importantly, the review emphasized that the long-term effects of thinning on soil physical structure (e.g., aggregate stability) are key determinants of whether carbon can be effectively sequestered. In well-drained stands with moderate soil texture, moderate thinning often enhances soil carbon stocks through increased physical protection; whereas on poor or erosion-prone soils, high-intensity thinning may lead to a net loss of carbon pools. A knowledge synthesis by Mayer et al. (2020) further refined this perspective. They found that the effects of thinning on (SOC) stocks are highly dependent on initial stand conditions, thinning intensity, and subsequent residue management. For middle-aged stands experiencing intense competition and suppressed growth, low-to-moderate thinning (removing 15–30% of basal area) generally optimizes resource allocation, promotes the growth of residual trees and understory vegetation, and increases carbon inputs, while causing minimal soil disturbance—favoring the maintenance or even enhancement of SOC stocks. However, high-intensity thinning (> 40%), although rapidly increasing light and water availability in the short term, may also cause a sharp increase in soil respiration rates and accelerated decomposition of existing organic matter due to the substantial removal of biomass and drastic alterations to the microclimate. The net effect is often uncertain and, in some cases, negative.

Synthesizing these studies, it becomes evident that the contradictory conclusions in the existing literature do not stem from methodological errors, but rather reflect the highly context-dependent nature of thinning effects. The innovation of the present study lies precisely in its transcendence of the simple binary conclusion of “increase” or “decrease,” to instead delve into how thinning affects carbon storage through the cascading pathway of regulating “soil physicochemical properties and organic carbon.” For example, *Jaehyun Lee* et al. research (Lee et al., 2024) clearly indicated that thinning enhances soil carbon storage capacity by shifting the soil environment toward an oligotrophic state. Huang and Zhou (2024) provided direct evidence that in large Chinese fir timber forests, the influence of thinning on soil carbon storage is mediated by changes in soil physicochemical properties and microbial community composition. Collectively, these findings converge on a core mechanism: thinning does not directly affect total carbon stocks, but rather, by reshaping the soil micro-ecosystem, it alters carbon stabilization pathways—such as promoting the transformation of microbial necromass into mineral-associated organic matter. Therefore, future research should pay greater attention to the correlation between soil properties and carbon stocks, rather than focusing solely on changes in carbon “quantity.” This represents a critical step toward resolving current debates and optimizing forest carbon sink management.

Near-infrared spectroscopy (NIRS) technology, known for its rapid and non-destructive analysis, has found extensive application in the detection of soil carbon and nitrogen (Wu et al., 2021; Ruma et al., 2024; Hu and Viscarra Rossel, 2025). This technique utilizes the molecular vibration spectra of hydro-gen-containing groups (C-H, O-H, N-H) to accurately predict SOC levels, exemplified by a coefficient of determination (*R*²) of 0.964 for black soil (Geng et al., 2024; Zheng et al., 2024). In the forestry sector, NIRS has been effectively employed for wood property analysis and has gradually expanded to include forest soil carbon monitoring in recent years, with a BPNN prediction yielding an *R*² of 0.849 (Zhang et al., 2021; Lin et al., 2024a). Nevertheless, model accuracy remains due to variations in sample selection and spectral preprocessing (Zhang et al., 2023; Ruma et al., 2024). Furthermore, research integrating thinning interventions with NIRS modeling to assess soil carbon dynamics is still limited (Harsányi et al., 2024; Zheng et al., 2024). This integration is necessary because it enables rapid, non-destructive assessment of soil carbon responses to management interventions across large spatial scales where traditional wet chemistry would be prohibitively time-consuming and costly, while also providing a methodological framework for developing predictive tools to support adaptive forest management decisions without repeated destructive sampling.

This study examines the soil of larch-birch natural secondary forests in the Greater Khingan Mountains following tending thinning. The objectives are: (1) to investigate the effects of varying thinning intensities on soil physicochemical properties and carbon storage, and to elucidate the associtions between tending intensity and soil carbon storage through correlation analysis, thereby identifying the thinning associated with the highest carbon storage under the conditions of this study; and (2) to develop a SOC content prediction model using NIRS and BPNN, which will serve as an effective monitoring tool for managing forest soil carbon sinks. The findings will offer both theoretical and practical foundations for the sustainable management of secondary forests and responses to climate change. This study integrates thinning intensity gradient experiments with NIRS-BPNN modeling to reveal the patterns of carbon dynamics and develop rapid detection methods.

## 2 Materials and methods

### 2.1 Overview of the study area

The study area is located in the *Xinlin* District on the northeastern slope of the *Yilehuli* Mountains in the northern part of Heilongjiang Province (51°20’–52°10’ N, 123°41’–125°25’ E), with a total area of 8702.94 km^2^ and a forest coverage rate of 84%. The study plots are situated in the *Xinlin* Forest Farm (143926 ha), with an average elevation of approximately 600 m and gentle terrain (slope< 6°). The area has a cold temperate continental climate, with an average annual temperature of -2.6 °C and annual precipitation of 513.9 mm (70% concentrated in July and August), and a frost-free period of only 90 days. The soil is mainly acidic mountainous brown coniferous forest soil (pH ≈ 5), with a thickness of 15–30 cm, and the humus content in the topsoil reaches 20%, but the gravel content is 30%–40%.

The vegetation is a typical natural mixed secondary forest of conifers and broad-leaved trees, with dominant tree species including Dahurian larch (*Larix gmelinii*) and white birch (*Betula platyphylla*). The understory is dominated by bush clover (*Lespedeza bicolor*) and Daurian rhododendron (*Rhododendron dauricum*). In 2007, twenty 100m×100m fixed permanent plots were established in compartments 106–109 with consistent slope(6°). Thinning teratments ranging from 0% to 59.9% were implemented, harvesting mature larch individuals. The residues were treated by composting, and unthinned control plots were retained. This area represents a typical plot for the study of management and carbon cycling of natural secondary forests in China’s cold temperate zone.

**Figure 1:**
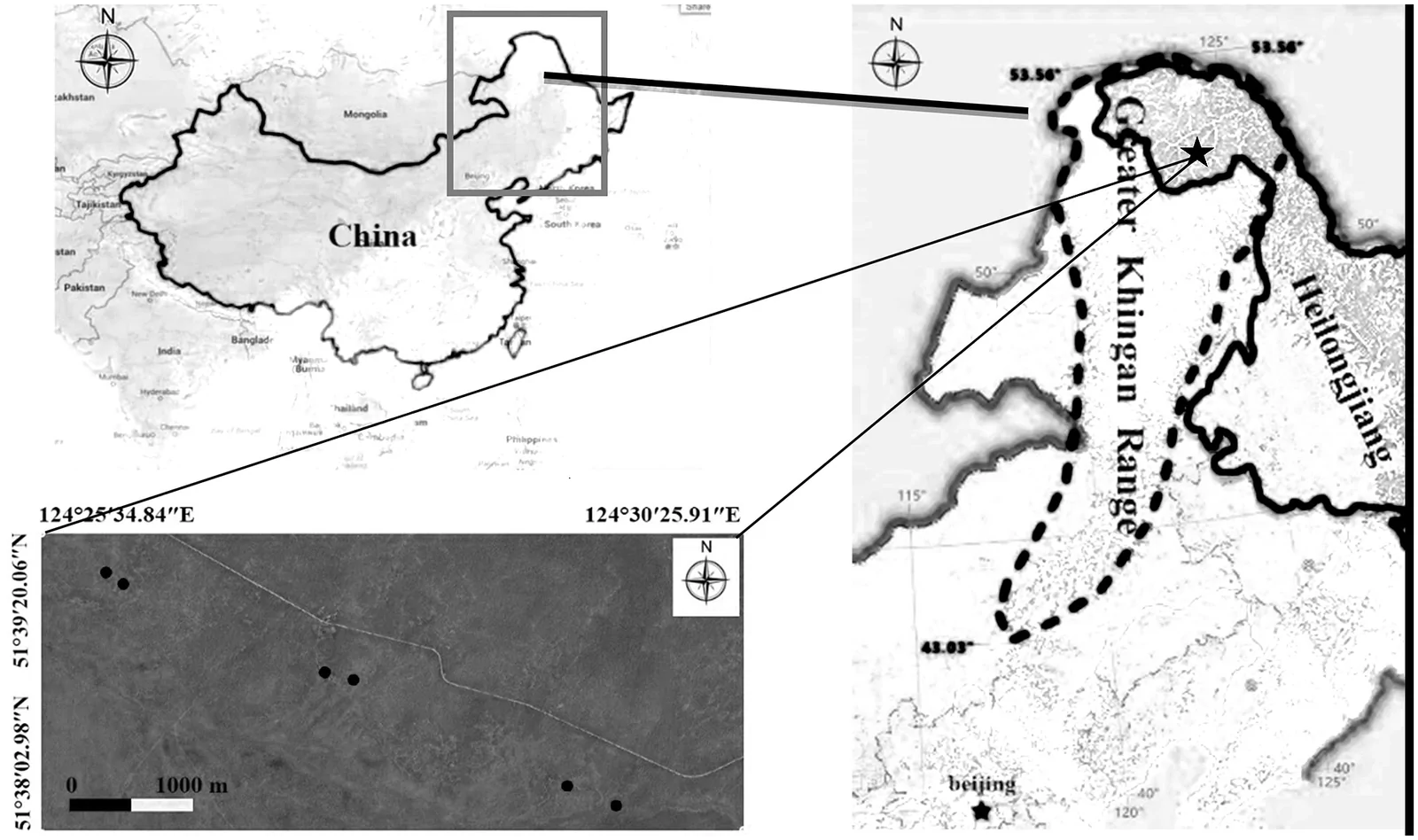
The geographical location of the study area and sample plots.

A map of the study area showing the geographic location and distribution of sample plots is prepared as **Figure 1**. The fundamental stand characteristics for each thinning treatment (stand density, mean DBH, mean tree height, and stand age) are provided in **Table 1**.

**Table 1:** Basic situation of thinning sample plots.

Thinning intensity	Stand age	Stand retention density (trees/ha)	Mean DBH (cm)	Mean height (m)
CK	Middle-aged	2175	8.34	9.15
16.7%	Middle-aged	2000	6.96	10.33
25.5%	Middle-aged	1675	7.06	9.69
34.4%	Middle-aged	1050	6.19	7.68
49.6%	Middle-aged	925	7.61	10.48
59.9%	Middle-aged	700	7.06	9.35

### 2.2 Plot setting and investigation

Taking the natural secondary forest of larch and birch as the research object, we selected six large-span thinning intensity groups from the 20 thinning gradients under the aforementioned management background to explore the association between thinning intensity and forest carbon storage. The selected thinning intensities were 0%, 16.7%, 25.5%, 34.4%, 49.6% and 59.9% respectively. Among them, 0% served as the control plot (unthinned), denoted as Group CK. To reduce errors, three 20 m × 20 m test plots were randomly established within each 100 m × 100 m plot as replicates, with at least 10 m buffers between subplots to ensure independence., The subplots did not overlap with each other.

### 2.3 Determination of soil carbon storage

**Figure 2:**
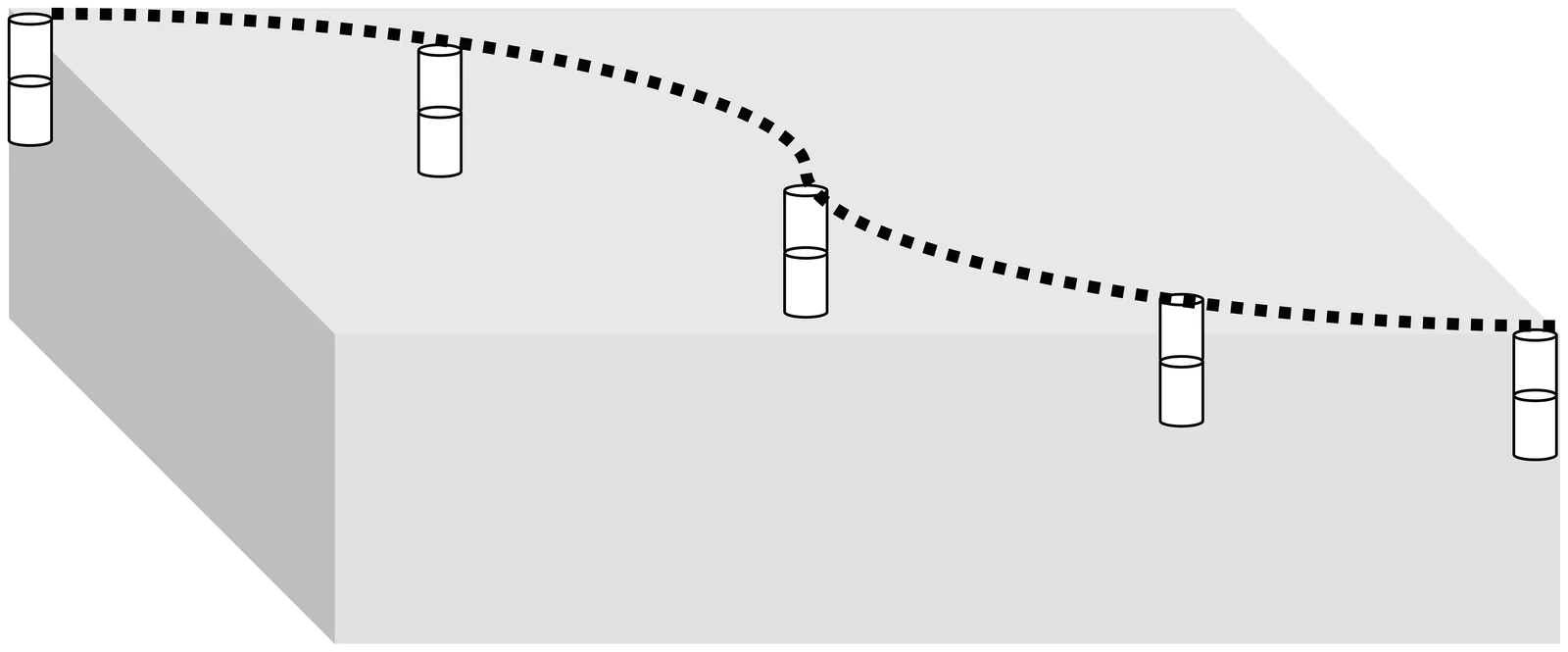
Distributions of soil sample.

Five sample points were arranged in an “S” shape within each plots (as shown in **Figure 2**). After removing debris from the upper layer of the soil at the sample points, soil samples were collected from the 0–10 cm and 10–20 cm soil layers using a stainless steel ring knife with a volume of 100 cm^3^. These samples were brought back to the laboratory and processed separately. For each individual sample point, the soil sample was dried in an 80 °C oven until a constant weight was achieved to determine the bulk density. Additionally, approximately 1 kg of soil was collected from each sample point for chemical analysis. These samples were air-dried naturally, with animal and plant residues and crushed stones removed, and then ground and sieved (0.25 mm)for SOC content determination using the potassium dichromate oxidation method (as described in Section 2.4.2). The carbon storage for each of the five sample points was calculated individually using the measured bulk density and organic carbon content. The final soil carbon storage value for each plot and soil layer was then obtained by averaging the five independent measurements. This approach ensured that within-plot spatial variability was accounted for in our analysis. It should be emphasize that the soil carbon storage values reported in this study were calculated using measured SOC values, not NIRS-predicted values. The NIRS-BPNN model was developed independently for future rapid prediction applications. The soil carbon storage density was calculated from soil bulk density, measured SOC content and the thickness of each layer.

The calculation formula for soil carbon storage per unit area [Equation (1)] is:

(1) $$CSi=\sum_{1}^{i}Ci\times Di\times Hi\times \left(1-Gi\right)/10$$

where *CSi* represents the carbon storage per unit area of the *i-*th layer of soil (t/ha), *Ci* represents the carbon content of the *i-*th layer of soil (g/kg), *Di* represents the bulk density of the *i-*th layer of soil (g/cm³), *Hi* represents the thickness of the *i-*th layer of soil (cm), *Gi* represents the proportion of gravel with a diameter of ≥2 mm in the *i-*th layer of soil (%), and 10 is the conversion factor per unit area.

### 2.4 Establishment of a model for detecting SOC content using near-infrared technology

#### 2.4.1 Near-infrared spectroscopy acquisition

The visible-near-infrared spectra (350–2500 nm) were collected using a LabSpec^®^ Pro FR/A114260 (Boulder Analytical Spectroscopy Equipment, USA), with spectral resolutions of 3 nm@700 nm and 10 nm@1400/2100 nm. To ensure the accuracy of the data, the experiment was conducted under dry conditions at room temperature. Before spectral collection, the spectrometer was preheated for 30 minutes and calibrated using a white board made of polytetrafluoroethylene. This plate reflects nearly 100% throughout the entire wavelength range (350–2500 nm). Whiteboard calibration was performed every 15 minutes. Each sample was scanned 30 times and automatically averaged into one spectrum. Each sample was measured three times, and the average spectrum was regarded as the original spectrum. The signal was generated in reflection mode and converted to absorbance using log(1/R).

#### 2.4.2 Chemical analysis of SOC

The samples to be tested were dried to a constant weight in a drying oven at a constant temperature of 85 °C. The samples were then ground and sieved (0.25 mm stainless steel sieve). The carbon content was determined and calculated by the potassium dichromate sulfuric acid wet heat oxidation method (LY/T1237-1999). During the experiment, the oil bath temperature was controlled at 170 to 180 °C, with a precise control time of 5 minutes. To ensure the accuracy of carbon content determination, each sample was measured three times, and the relative deviation of the three repetitions was controlled within 2%. If the average relative error exceeded 2%, the process was repeated, and the average of the three measurement results with the smallest difference was retained as the carbon content of the sample. The determination results of SOC content are shown in **Table 2**.

**Table 2:** Statistics of chemical determination results of the SOC content.

Parameter	Minimum (g/kg)	Maximum (g/kg)	Average (g/kg)	SD (g/kg)
SOC content	5.80	163.00	25.30	22.20

#### 2.4.3 Spectral data preprocessing

Due to the light scattering of solid samples and different effective path lengths, near-infrared spectral data inevitably contain some unnecessary variations or noise. To enhance model development and accuracy, it is necessary to preprocess the spectral data to reduce such unnecessary variations (Åsmund et al., 2009; Lin et al., 2017). In this study, seven important spectral pretreatment methods were adopted. First, the EMSC method was chosen. EMSC is an extension of the traditional MSC. It is not merely about removing multiplicative and additive effects from the spectrum. This extended method better separates the physical light scattering effect from the chemical light absorption effect by including wavelength-related effects or prior information in the modeling (Liu et al., 2010). Secondly, the OSC method was applied, which serves as the transformation method when establishing PLS regression models with spectral data. This method eliminates the irrelevant variance in the X data and sometimes makes the PLS model more accurate. Because OSC depends on the Y value, it requires a matrix with a Y value, which must be precise (Biney et al., 2021). Meanwhile, several commonly used spectral preprocessing methods were compared. For instance, spectral derivative preprocessing was performed through the gap derivatives of 27 points. Standardized preprocessing was performed using range standardization, where each row of the spectral matrix was divided by its range (maximum value). Baseline correction preprocessing was achieved by first running a linear baseline correction and then combining it through baseline offsets. Detrending preprocessing aimed to eliminate nonlinear trends in spectral data, with polynomial order 2 applied to the data.

#### 2.4.4 Construction of NIRS model for predicting SOC content based on BPNN

BPNN has self-learning, self-adaptative, and strong nonlinear function approximation capabilities. It can achieve near-infrared spectrum prediction functions and is a powerful tool for dealing with nonlinear regression. BPNN is a feedforward feedback neural network system, mainly composed of an input layer, a hidden layer and an output layer. Sequential transmission is adopted. Signals enter from the input layer, are processed by the hidden layer, and then output from the output layer. If the output result of the neural network’s output layer does not meet expectations, the signal is transmitted in reverse. According to the error value, the network threshold and weights are adjusted in reverse to make the predicted output of the network approach the expected output and minimize the error between the predicted and true values. The fundamental principles of BPNN are well-established in the literature (Xiang et al., 2014; Shikui, 2019). In this study, OSC-preprocessed spectral data (2,151 wavelengths) were used as inputs for BPNN modeling. The network architecture was determined through repeated experimental trials on the training set. We tested different numbers of hidden layer neurons (4, 6, 8, 10, 12, 15, 20) and evaluated model performance on the training set. The configuration with 2,151 input neurons (full spectral wavelengths), 12 hidden neurons, and 1 output neuron yielded the best balance between fitting accuracy and generalization capability. The activation function for the hidden layer was the sigmoid function, and the output layer used a linear activation function. The Levenberg-Marquardt algorithm was employed for network training.

Although using 2,151 wavelengths as inputs involves substantial computational cost, this configuration was selected because it retains the full spectral information. Due to the high collinearity among adjacent spectral wavelengths, the effective degrees of freedom are substantially lower than the nominal 2,151 variables, reducing the practical risk of overfitting. To further prevent overfitting, early stopping was applied: 20% of the training data was held out as an internal validation subset for monitoring generalization performance, and training was terminated when the validation error showed no improvement for 50 consecutive epochs. The BPNN was also trained multiple times with different random initializations to ensure stability.

In the division of the sample set, the original calibration set (48 samples) was used as the BPNN training set, and the validation set (24 samples) was used as the independent prediction set. The prediction set was strictly withheld from all stages of model development and was used only for final model performance evaluation.

#### 2.4.5 Model performance evaluation

Model quality assessment includes calibrated *R*-squared (*R*^2^c), predicted *R*-squared (*R*^2^p), calibrated root mean square error (RMSEC), cross-validated root mean square error (RMSECV), predicted root mean square error (RMSEP), and residual prediction bias (RPD). *R*^2^ is used to describe the linear correlation between predicted values and measured values. The higher the *R*^2^p and the closer the *R*^2^c is to 1, the greater the correlation between the predicted value and the actual value, and the stronger the robustness of the model. RMSEC, RMSECV and RMSEP are used to evaluate the feasibility of calibration models. The lower the RMSEP and the closer it is to RMSEC, the stronger the predictive ability of the calibration model. RPD is an indicator that measures the ability of a model to predict components. Values between 2.0 and 2.5 represent approximate quantitative predictions, while values between 2.5 and 3.0 and above 3.0 respectively indicate predictions that can be regarded as good and excellent (Li et al., 2015). The calculation formulas for these standards are as follows (Equations 2–4):

(2) $$R^{2}=1-\frac{{\sum_{i=1}^{n}\left(yi-\hat{ji}\right)}^{2}}{{\sum_{i=1}^{n}\left(yi-\hat{y}\right)}^{2}}$$

(3) $$RMSE=\sqrt{\frac{{\sum_{i=1}^{n}\left(yi-\hat{yi}\right)}^{2}}{n}}$$

(4) $$RPD=\frac{SD}{RMSEP}$$

In the formulas, *yi* represents the measured value of carbon content; $\hat{yi}$ and $\bar{y}$ represent the predicted value and average value of carbon content, respectively. n is the number of samples. When *n* is the number of samples in the calibration set, the determination coefficient and the root mean square error are respectively called *R*^2^c and RMSEC. When *n* is the number of samples in the validation set, the determination coefficient and the root mean square error are respectively called *R*^2^p and RMSEP. SD is the standard deviation of the measured carbon content value. Data statistical analysis was completed in IBM SPSS Statistics 26.0 (IBM, USA). The collected reflectance spectra were converted into absorbance spectrum data in ViewSpecPro software (ASD Corporation, USA). All spectral preprocessing was carried out in Unscramber^®^X v10.4 software (CAMO Software, USA). PLS modeling, BPNN modeling, and SPXY algorithm were implemented in MatlabR2016a (MathWorks, USA).

### 2.5 Determination of soil physical and chemical properties

1. Soil chemical properties: The chemical properties of 1 kg soil samples from 0–10 cm layer were determined, including pH value, total nutrient (N, P, K) content, and available nutrient (N, P, K) content. The indicators and determination methods are shown in **Table 3**.
2. Soil physical properties: The physical properties of the soil in the ring knife soil samples from the 0–10 cm soil layer were determined, mainly including five indicators: soil bulk density, maximum water-holding capacity, total porosity, capillary porosity, and non-capillary porosity. The specific procedure for the ring knife method is as follows: First, the upper cover of the ring knife was removed, and the mass was weighed using a precision balance, recorded as the mass of the soil sample inside the ring knife (*M0*). Then, the ring knife was placed in a flat-bottomed basin filled with clear water ensuring that the height of the water did not exceed the upper edge of the ring knife. After soaking for 12 hours, the water on the surface of the ring knife was dried and weighed, recording the wet weight of the soil inside the ring knife (*M12h*). Then, the ring knife was placed on dry sandy soil. After 2 hours, the mass was weighed and recorded as the weight of the wet soil inside the ring knife (*M2h*). The soil sample from the middle part of the ring knife was placed in an aluminum box and weighed, recording the mass (*Mw*) of the wet soil in the aluminum box. After that, the soil sample was dried at 105°C and the mass (*Md*) of the dried soil sample in the aluminum box was recorded. Based on the obtained results, the above indicators were calculated. The calculation formulas are as follows (Equations 5–13):

**Table 3:** Determination method of soil chemical properties.

Determination index	Determination method	National standard	Main instruments
pH Value	Water Extraction Method	LY/T1239—1999	pH Meter
Total Nitrogen (TN)	Automatic Kjeldahl Method	LY/T1228—1999	Automatic Nitrogen Analyzer
Total Phosphorus (TP)	Acid Dissolution-Molybdenum Antimony Antispectrophotometric Method	LY/T1232—1999	Atomic Absorption Spectrophotometer
Total Potassium (TK)	Carbon Dissolution-Flame Photometric Method	LY/T1234—1999	Flame Photometer
Available Nitrogen (AN)	Alkaline Hydrolysis Diffusion Method	LY/T1231—1999	Diffusion Dish, Constant Temperature Oven
Available Phosphorus (AP)	Sodium Hydroxide Extraction-Molybdenum Antimony Antispectrophotometric Method	LY/T1233—1999	Atomic Absorption Spectrophotometer
Available Potassium (AK)	Ammonium Acetate Extraction-Flame Photometric Method	LY/T1236—1999	Flame Photometer

(5) $$R0=\frac{Mw-Md}{Md}\times 100\%$$

(6) $$RM^{\prime }=M0\times K$$

(7) $$D=\frac{M^{\prime }}{V}$$

(8) $$K=\frac{Md}{Mw}$$

where *R_0_* represents the soil moisture content, *M’* represents the mass of the dried soil sample, *K* is the conversion factor, *D* is the soil bulk density, and *V* is the ring knife volume (100 cm^3^).

(9) $$M\max=\frac{M12h-M2h}{M2h}\times 100\%$$

(10) $$Mc=\frac{M2h-M^{\prime }}{M^{\prime }}\times 100\%$$

(11) Pnc=(Mmax−Mc)×D×100%

(12) Pc=Mc×D×100%

(13) Pt=Pc+Pnc

where *M*max and *Mc* represent the maximum water-holding capacity and capillary water-holding capacity of the soil, respectively, while *Pt*, *Pc*, and *Pnc* represent the total porosity, capillary porosity, and non-capillary porosity of the soil, respectively.

## 3 Results

### 3.1 Soil near-infrared spectral characteristics and model development

#### 3.1.1 Analysis of soil near-infrared spectral characteristics

**Figure 3:**
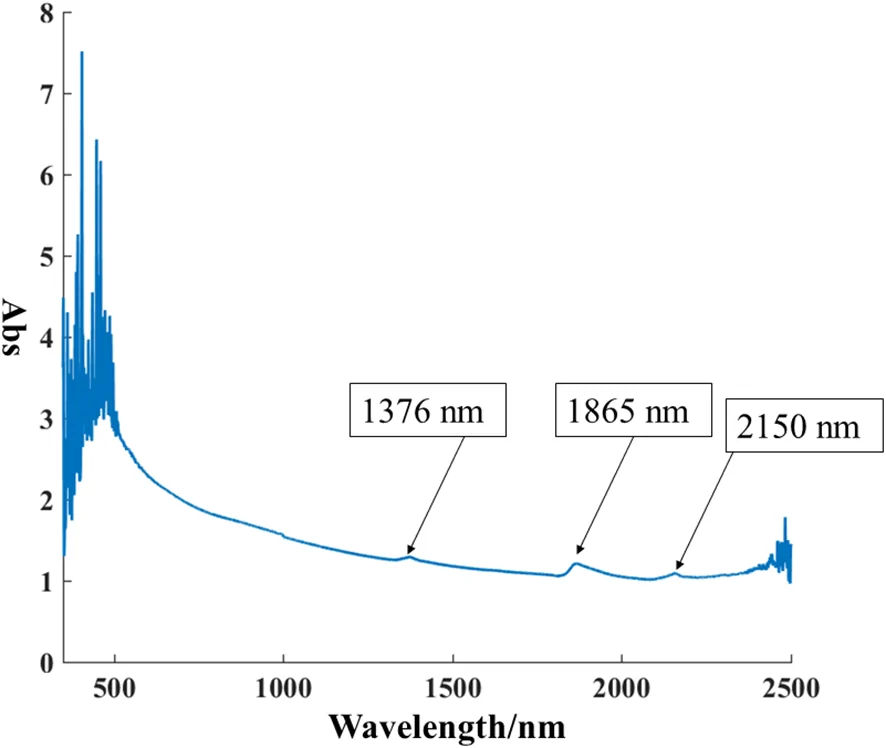
The Vis-NIR spectra analysis of the soil sample.

The vision-near-infrared spectra (350–2500 nm) of the soil samples are generally relatively flat, with peaks occurring around 1376 nm, 1865 nm, and 2150 nm, as shown in **Figure 3**. The peak at 1376 nm is generated by the combination of C-H stretching vibration and bending vibration. The peak near 1865 nm is related to the first-order overtone of the stretching vibrations of O-H and C-H. The peak at 2150 nm represents the combination of bending vibration in the N-H plane and stretching vibrations of C-H and C=O. The peak positions of the near-infrared spectral characteristics of the soil are very similar to those in previous studies (Cozzolino and Morn, 2003; Shen et al., 2010; Li et al., 2014), all occurring around these three wavelengths. The slight differences in wavelength positions are due to the different types of soil. In the near-infrared spectra of soil samples, the intensity of absorption peaks is relatively weak. Therefore, it is necessary to enhance the effective information in the spectra through preprocessing, improve the signal-to-noise ratio, and thereby enhance the performance of the model.

#### 3.1.2 Division of the sample set

Before preprocessing the spectra, taking the measured SOC content as the Y variable and the spectral data as the X variable, the sample set was divided using the SPXY algorithm. A total of 72 samples were divided into 48 calibration sets and 24 validation set samples in a ratio of 2:1 using the SPXY algorithm. The statistical results are shown in **Table 4**. In **Table 4**, the carbon content of the calibration set samples covers the carbon content range of the validation set samples, and the coefficient of variation is 95.31%, which is higher than that of the validation set. The degree of dispersion of the calibration set is relatively large, which will be conducive to the accurate prediction of unknown samples by the model.

**Table 4:** Statistical results of carbon content in the validation set and calibration set.

Sample set	Number of samples	Minimum (g/kg)	Maximum (g/kg)	Average (g/kg)	SD (g/kg)	Coefficient of variation (%)
calibration set	48	5.8	163.0	27.67	26.37	95.31
validation set	24	5.8	38.4	19.67	9.84	50.02

#### 3.1.3 Selection of soil near-infrared spectroscopy pretreatment methods

After preprocessing the spectral data and determining the corresponding optimal number of principal components, a PLS regression model was established. With the original spectra as the control, the best preprocessing method was selected. The result statistics are shown in **Table 5**. Compared with the results of the original spectrum, not all pretreatment methods improved the model’s results. Derivative preprocessing of spectral data had no effect on the model improvement. Other preprocessing methods improved model performance to varying degrees. After EMSC preprocessing, the *R*^2^c of the model correction set increased by 8.6%, while OSC showed the best effect on improving model performance. Compared with the Raw model, the *R*^2^c of the correction set increased by 22.2%, and the *R*^2^p of the validation set increased by 21.5%, yielding better model performance than the original spectrum and other preprocessing methods. After OSC processing, the model evaluation parameters *R*^2^c and *R*^2^p were 0.8683 and 0.8726, respectively. EMSC pretreatment enhanced the performance of soil models and eliminated scattering caused by uneven particles in soil samples. OSC preprocessing significantly improved model performance by combining the Y variable with the X variable and eliminating irrelevant information in the X variable.

**Table 5:** PLS regression models of SOC content based on different pretreatments.

Spectral preprocessing method	Five-fold cross-validation	Calibration set		Validation set	
	OPC	*R*^2^c	RMSEC	*R*^2^p	RMSEP
Raw	2	0.7112	0.0138	0.7180	0.0051
EMSC	4	0.7726	0.0123	0.7848	0.0034
1D	1	0.6334	0.0156	0.5862	0.0062
2D	1	0.4650	0.0188	0.5473	0.0065
Baseline correction	4	0.7431	0.0130	0.8087	0.0039
de-trend	2	0.7231	0.0140	0.7087	0.0057
OSC	6	0.8683	0.0093	0.8726	0.0028
normalization	3	0.7415	0.0131	0.7577	0.0036

#### 3.1.4 Establishment and performance of the BPNN model for predicting SOC

The BPNN differs from PLS in that it performs nonlinear regression and has the advantage of adaptive learning. OSC-preprocessed spectral data (2,151 wavelengths) were used as inputs for BPNN modeling. The network architecture was determined through repeated experimental trials on the training set. We tested different numbers of hidden layer neurons (4, 6, 8, 10, 12, 15, 20) and evaluated model performance on the training set. The configuration with 2,151 input neurons (full spectral wavelengths), 12 hidden neurons, and 1 output neuron yielded the best balance between fitting accuracy and generalization capability. The activation function for the hidden layer was the sigmoid function, and the output layer used a linear activation function. The Levenberg-Marquardt algorithm was employed for network training.

Although using 2,151 wavelengths as inputs involves substantial computational cost, this configuration was selected because it retains the full spectral information. Due to the high collinearity among adjacent spectral wavelengths, the effective degrees of freedom are substantially lower than the nominal 2,151 variables, reducing the practical risk of overfitting. To further prevent overfitting, early stopping was applied: 20% of the training data was held out as an internal validation subset for monitoring generalization performance, and training was terminated when the validation error showed no improvement for 50 consecutive epochs. The BPNN was also trained multiple times with different random initializations to ensure stability of the results.

**Figure 4:**
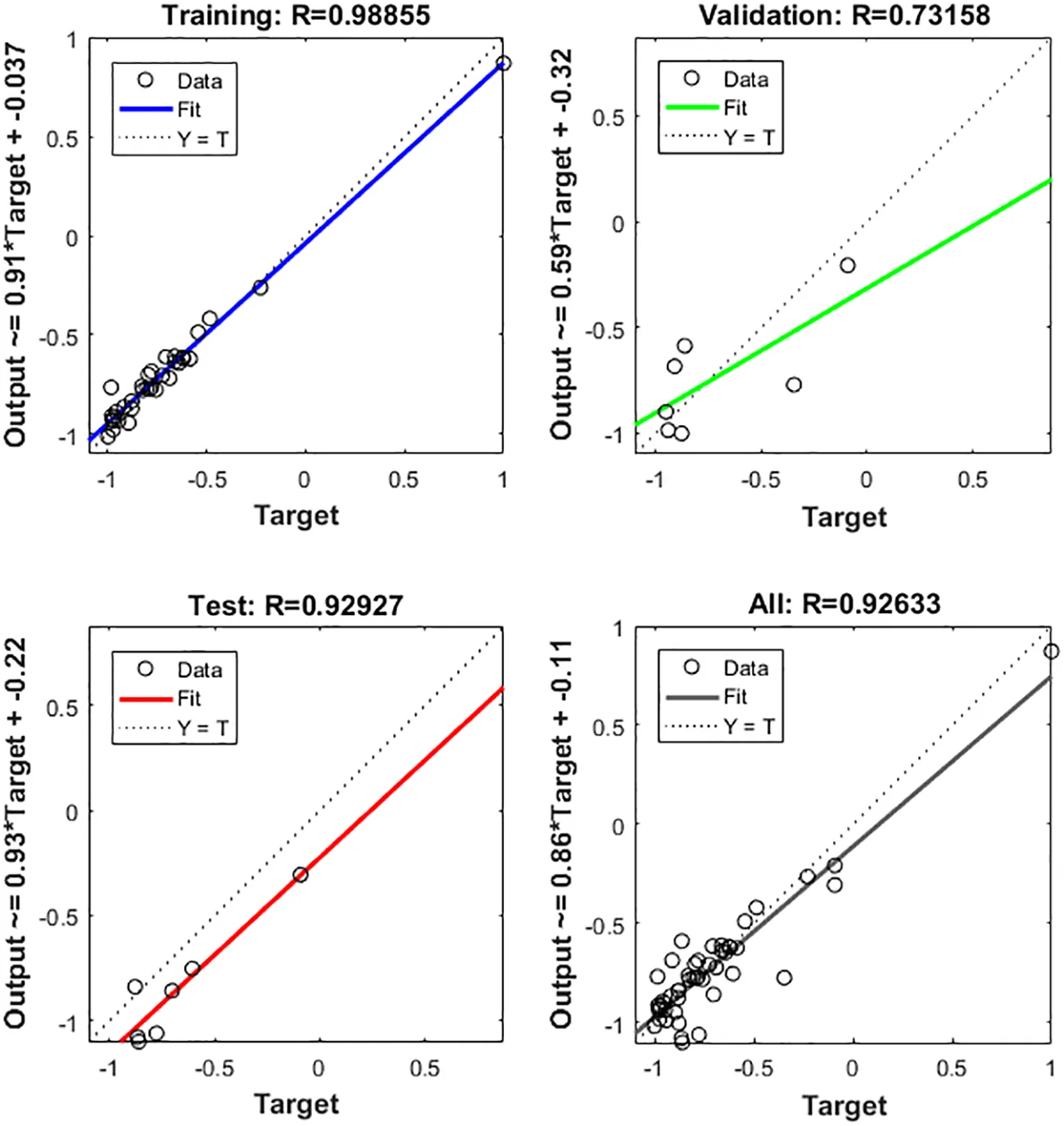
BP model parameters and regression curves.

In the division of the sample set, the original calibration set (48 samples) was used as the BPNN training set, and the validation set (24 samples) was used as the independent prediction set. The prediction set was strictly withheld from all stages of model development and was used only for final model performance evaluation. The correlation coefficients and results of the models are shown in **Figure 4**. The regression coefficient *R* of the training set is 0.9886, and that of the prediction set was 0.9293, indicating that the established model has good performance. The full performance metrics for the BPNN model were: *R²c* = 0.9773, RMSEC = 0.0030, *R²p* = 0.8635, RMSEP = 0.0064, and RPD = 2.8, indicating good predictive performance. The strong performance on the fully independent prediction set (R = 0.9293, RMSEP = 0.0064) provides empirical evidence for good generalization. The model was trained with early stopping to prevent overfitting, and repeated training with different random initializations confirmed the stability of the results.

**Figure 5:**
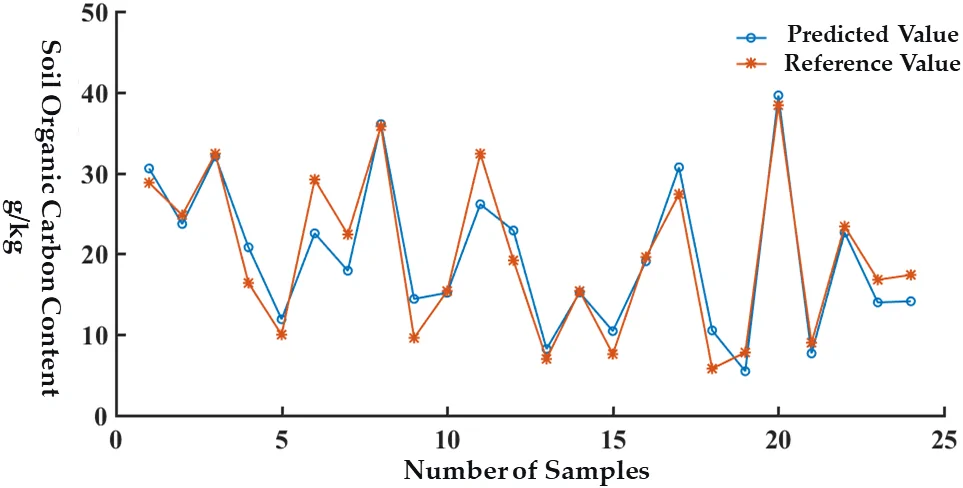
Comparison of SOC content between test results and prediction results.

The performance of the soil near-infrared model in this study is superior to the research results of *Seema* (Seema et al., 2020), *S. Nawar* (Nawar and Mouazen, 2019), *Dotto* (Dotto et al., 2018), etc. established a SOC prediction model by comparing partial least squares regression (PLSR), random forest (RF), support vector regression (SVR), and multivariate adaptive regression (MARS), and found that the PLSR model had the best performance, but its *R*^2^ was only 0.73. *S. Nawar* established a near-infrared model using RF, with a coefficient of determination *R*^2^ value of 0.84. *Dotto* applied ANN to establish a near-infrared prediction model for SOC content (*R*^2^ = 0.80, RMSE = 0.51). *Viscarra Rossel* et al. ‘s research found that compared with PLSR, MLR, SVM and RF, ANN achieved the best prediction results of SOC, with an *R*^2^ of 0.89 and an RMSE of 0.75% (Rossel and Behrens, 2010), which is relatively close to the performance of the BPNN model in our research. The excellent performance of the BPNN model may be attributed to its characteristics in solving nonlinear problems (Mouazen et al., 2015). In the BPNN, the mathematical model allocates weights among elements, and the network structure is adjusted according to the input (Mcbratney et al., 2003). The performance of the various soil near-infrared spectroscopy prediction models mentioned above varies due to many factors, such as soil type, soil particle size, soil sample set size, the selection of near-infrared spectroscopy pretreatment methods, and the choice of modeling methods. In this study, the OSC pretreatment method was adopted, and a near-infrared model for predicting SOC content was established through the BPNN. The research results show that the model established through BPNN has a good prediction effect on SOC content. The predicted values of the model have a high correlation with the values determined by laboratory chemical methods (*R* = 0.9293) (as shown in **Figures 4**, **5**), meeting the accuracy requirements for predicting SOC content and facilitating the realization of large-scale SOC content detection in secondary forests.

### 3.2 Effects of tending thinning intensity on soil physicochemical properties

#### 3.2.1 Impact of tending thinning on soil physical properties

The physical properties of soil under different tending thinning intensities and the results of their statistical analysis are presented in **Table 6**.

**Table 6:** Soil hydrological performance of different thinning intensity plots.

Thinning intensity	Moisture content (%)	Soil bulk density (g/cm3)	Maximum water-holding capacity (%)	Total porosity (%)	Capillary porosity (%)	Non-capillary porosity (%)
CK	33.02 ± 2.13a	1.31 ± 0.14a	61.18 ± 8.93a	52.24 ± 11.85a	42.12 ± 7.88a	10.11 ± 4.87a
16.7%	25.00 ± 6.00a	1.52 ± 0.18b	93.35 ± 11.25bc	66.23 ± 10.57a	51.98 ± 10.22ab	14.32 ± 4.63b
25.5%	23.67 ± 4.62a	1.64 ± 0.11b	110.05 ± 13.56c	72.16 ± 14.77a	57.36 ± 9.24b	15.19 ± 6.82b
34.4%	56.67 ± 5.54b	1.29 ± 0.25a	92.54 ± 14.28bc	66.58 ± 13.68a	52.76 ± 8.97ab	12.96 ± 3.29ab
49.6%	22.67 ± 6.1a	1.32 ± 0.17a	97.32 ± 10.33c	52.23 ± 11.79a	42.36 ± 7.65a	10.42 ± 3.81a
59.9%	29.33 ± 4.16a	1.39 ± 0.06b	84.55 ± 10.23b	48.24 ± 12.22a	39.47 ± 6.43a	9.31 ± 2.38a

Different letters in the same column indicate significant differences among different thinning groups at the same soil layer (*p<* 0.05), while the same letter indicates no significant differences(*p* > 0.05).

As shown in **Table 6**, after treatments with different thinning intensities, the soil moisture content was highest in the 34.4% thinning group, followed by the CK group. No clear pattern of change in soil moisture content was observed with increasing thinning intensity. The soil moisture content in the 34.4% thinning group was significantly higher than that in the CK group and the other thinning groups (*p<* 0.05).

Soil bulk density initially increased, then decreased, and then increased again with rising thinning intensity, reaching its minimum value at the 34.4% thinning intensity. Although there was no significant difference compared to the CK group and the 49.6% thinning group, it was significantly lower than that in the 16.7%, 25.5%, and 59.9% thinning groups.

The maximum water-holding capacity of the soil showed no consistent pattern with increasing thinning intensity. It was highest in the 25.5% thinning group and lowest in the control group. Notably, the maximum water-holding capacity in all thinning groups was significantly higher than that in the CK group (*p<* 0.05).

Total porosity initially increased and then decreased with increasing thinning intensity, peaking at the 25.5% thinning intensity and reaching its lowest value at the 59.9% thinning intensity. However, no significant differences in total porosity were observed among the various groups (*p* > 0.05).

Similarly, capillary porosity followed the same trend as total porosity with increasing thinning intensity, reaching its highest value at the 25.5% thinning intensity. It differed significantly from the 49.6% and 59.9% thinning groups as well as the CK group (*p<* 0.05).

Non-capillary porosity also exhibited a trend similar to that of total porosity, peaking at the 25.5% thinning intensity. The CK group and the high-intensity thinning groups (49.6% and 59.9%) showed lower non-capillary porosity, with no significant differences among them (*p* > 0.05). However, they were significantly lower than the 16.7% and 25.5% thinning groups (*p<* 0.05).

#### 3.2.2 The influence of tending thinning intensity on soil chemical properties

The chemical properties of the soil in the sample plots with different tending and thinning intensities and the analysis results of their differences are shown in **Table 7**.

**Table 7:** Soil chemical properties of plots with different thinning intensities.

Thinning intensity	pH	Soil organic matter (g/kg)	Mass fraction of total nutrients (g/kg)			Mass fraction of available nutrients (mg/kg)		
			Total N	Total P	Total K	Available N	Available P	Available K
CK	5.19 ± 0.16ab	55.22 ± 15.1 a	1.56 ± 0.14ab	0.64 ± 0.09a	18.38 ± 3.70a	165.78 ± 20.25a	21.49 ± 2.82a	175.62 ± 25.03a
16.7%	5.03 ± 0.14a	30.05 ± 15.32 a	1.45 ± 0.20a	0.71 ± 0.08ab	22.63 ± 2.05ab	245.97 ± 26.70b	28.06 ± 3.29a	168.91 ± 20.22a
25.5%	5.39 ± 0.16b	23.15 ± 11.20 a	1.88 ± 0.11c	0.97 ± 0.11c	29.11 ± 2.96c	286.05 ± 26.18c	40.2 ± 4.06bc	285.47 ± 25.44c
34.4%	5.74 ± 0.23c	114.08 ± 28.04 b	1.76 ± 0.12bc	0.86 ± 0.09bc	24.37 ± 3.19b	302.15 ± 27.21c	43.87 ± 4.86c	302.38 ± 22.13c
49.6%	4.79 ± 0.20a	33.84 ± 9.62 a	1.34 ± 0.18a	0.82 ± 0.12bc	18.6 ± 3.17a	216.01 ± 18.85ab	31.45 ± 3.66ab	344.66 ± 28.68c
59.9%	5.33 ± 0.18b	36.72 ± 10.28 a	1.57 ± 0.16ab	0.75 ± 0.14ab	20.74 ± 2.84ab	189.2 ± 31.38ab	35.76 ± 5.03b	214.72 ± 30.78b

Different letters in the same column indicate significant differences among different thinning groups (*p* < 0.05), while the same letter indicates no significant differences (*p* > 0.05).

The differences in soil chemical properties among various plots: As shown in **Table 7**, with increasing of thinning intensity, the variation pattern of soil pH was not obvious. However, at 34.4% thinning intensity, the pH of the sample plots was significantly higher than that of the CK group and other thinning groups (*p<* 0.05), while there was no significant difference between the CK group and the other groups except the 34.4% thinning intensity group (*p* > 0.05). Soil organic matter content showed no obvious variation pattern with increasing thinning intensity. At 34.4% thinning intensity, the organic matter content reached its highest value, while at 25.5% thinning intensity, it was the lowest. The difference in organic matter content between the two plots was as high as 53g/kg. The organic matter content in the 34.4% thinning intensity group was significantly higher than that in the CK group and other thinning group (*p<* 0.05). Total soil nutrients showed a trend of first increasing and then decreasing with the increasing thinning intensity. At 25.5% and 34.4% thinning intensities, the contents of total N, total P and total K in the soil were significantly higher than those in the CK group (*p<* 0.05), and the content of total K was significantly higher than that in the CK group and other thinning groups (*p<* 0.05). Available nutrients in the soil generally showed a similar trend to the total nutrients with increasing thinning intensity, first increasing and then decreasing. Available N and available P contents reached the maximum value at 34.4% thinning intensity and were significantly higher than those in the CK group (*p<* 0.05). The available K content reached its maximum at 49.6%, and there was no significant difference compared with the 25.5% and 34.4% intensity thinning groups (*p* > 0.05). The available K content in the CK, 16.7%, and 59.9% thinning groups was significantly lower than that in the 25.5%, 34.4%, and 49.6% thinning groups (*p<* 0.05). Overall, both total and available nutrient contents increased to varying degrees compared with the CK group when thinning intensity exceeded 25.5%.

### 3.3 The influence of tending and thinning intensity on soil carbon storage

#### 3.3.1 The influence of tending and thinning intensity on SOC content

**Table 8** shows that the SOC in the 0–10 cm soil layer with different thinning intensities exhibited no obvious change pattern with increasing thinning intensity. However, the SOC content in the 34.4% thinning group was significantly higher than that in the CK group and the other thinning groups (*p<* 0.05). In the 0–10 cm soil layers, among the five thinning groups, carbon content reached its maximum at 34.4% thinning intensity, increasing by 34.14 g/kg compared with the CK plot, more than twice that of the CK plot, significantly improving the SOC content. Compared with the CK group, the SOC content in other intensity thinning groups was lower. The variation pattern of organic carbon content in the 10–20 cm soil layer was not obvious. The carbon content in the 25.5%thinning group was 4.34 g/kg higher than that in the control group, while that in the 49.4% thinning group was only 1.14 g/kg higher. The carbon content in the other three thinning groups was lower than that in the control plot.

**Table 8:** SOC content under different thinning intensities.

Thinning intensity	SOC content (g/kg)	
	0~10 cm	10~20 cm
CK	32.03 ± 5.03 b*	13.03 ± 6.51 a
16.7%	17.43 ± 6.25 b	9.83 ± 3.72 a
25.5%	13.43 ± 7.70 b	17.37 ± 9.87 a
34.4%	66.17 ± 14.84 a	10.23 ± 4.58 a
49.6%	19.63 ± 9.82 b	14.17 ± 8.17 a
59.9%	21.30 ± 5.29 b*	9.17 ± 1.00 a

Different letters in the same column indicate significant differences among different thinning groups at the same soil layer (*p<* 0.05), while the same letter indicates no significant differences among different thinning groups at the same soil layer (*p* > 0.05); * indicates significant differences between different soil layers within the same thinning group (*p<* 0.05).

It can also be seen from **Table 8** that, except for the 25.5% thinning group, carbon content of the 0–10 cm soil layer in the same plot of the other thinning groups was higher than that of the 10–20 cm soil layer. The organic carbon content in the 0–10 cm soil layer was approximately twice that of the 10–20 cm soil layer. The organic carbon content in the 0–10 cm and 10–20 cm soil layers was significantly different in the CK group and the 59.9% thinning group (*p<* 0.05).

#### 3.3.2 The influence of tending and thinning intensity on soil bulk density

**Table 9** shows that the soil bulk density in the 0–10 cm and 10–20 cm layers showed significant differences (*p<* 0.05) among the thinning groups of different intensities, but there was no obvious variation pattern among the different thinning groups. In the 0–10 cm soil layer, except for the 34.4% intensity thinning group, which had a lower bulk density than the control group, the bulk densities of the other thinning groups were all higher than that of the control group. In the 10–20 cm soil layer, bulk density in the thinning groups was generally lower than that in the control group.

**Table 9:** Soil bulk density at different thinning intensities.

Thinning intensity	Soil bulk density (g/cm^3^)	
	0~10 cm	10~20 cm
CK	1.31 ± 0.14 b	1.68 ± 0.18 ab
16.7%	1.52 ± 0.18 a	1.62 ± 0.19 ab
25.5%	1.64 ± 0.11 a	1.46 ± 0.24 b
34.4%	1.29 ± 0.25 b*	1.83 ± 0.12 a
49.6%	1.32 ± 0.17 b	1.48 ± 0.06 b
59.9%	1.39 ± 0.06 a*	1.68 ± 0.04 ab

Different letters in the same column indicate significant differences among different thinning groups at the same soil layer (p< 0.05), while the same letter indicates no significant differences (p > 0.05); * indicates significant differences between different soil layers within the same thinning group (p< 0.05).

It can also be seen from **Table 9** that in the same thinning group, bulk density in the 0–10 cm soil layer was generally lower than that in the 10–20 cm soil layer, indicating that within the same plot, soil bulk density increased with soil depth, except for the 25.5% thinning group. In the 34.4% and 59.9% thinning groups, there was a significant difference in soil bulk density between the 0–10 cm and 10–20 cm soil layers (*p<* 0.05).

#### 3.3.3 The influence of tending and thinning intensity on soil moisture content

**Table 10** shows that in the 0–10 cm soil layer, 34.4% thinning intensity significantly increased the soil moisture content (*p<* 0.05). The soil moisture content in the 10–20 cm layer showed no significant overall difference among the thinning groups of different intensities (*p* > 0.05). The soil moisture content of different thinning groups in the 0–10 cm soil layer was generally lower than that of the CK group. However, the soil moisture content in the 34.4% thinning group was higher than that in the control group, while the moisture content in the other thinning groups was lower than that in the control group. In the 10–20 cm soil layer, the soil moisture content in all five thinning groups was all higher than that in the control group, contrary to the trend in the 0–10 cm layer. However, there was no obvious change pattern among the different intensity thinning groups.

**Table 10:** Soil moisture content at different thinning intensities.

Thinning intensity	Moisture content (%)	
	0~10 cm	10~20 cm
CK	33.02 ± 2.13 a*	17.67 ± 1.15 a
16.7%	25.00 ± 6.00 a	20.33 ± 4.62 a
25.5%	23.67 ± 4.62 a	23.33 ± 3.51 a
34.4%	56.67 ± 5.54 b*	19.00 ± 4.58 a
49.6%	22.67 ± 6.1 a	20.00 ± 4.00 a
59.9%	29.33 ± 4.16 a	24.67 ± 2.08 a

Different letters in the same column indicate significant differences among different thinning groups at the same soil layer (*p* < 0.05), while the same letter indicates no significant differences (*p* > 0.05); * indicates significant differences between different soil layers within the same thinning group (*p* < 0.05).

It can also be seen from **Table 10** that in the same thinning group, the moisture content in the 0–10 cm soil layer was generally higher than that in the 10–20 cm soil layer, indicating that within the same plot, soil moisture content decreased with soil depth. In the 34.4% thinning and CK groups, there was a significant difference in soil moisture content between the 0–10 cm and 10–20 cm soil layers (*p<* 0.05).

#### 3.3.4 The influence of thinning intensity on carbon storage in different soil layers

**Table 11** shows that under different thinning intensities, in the 0–10 cm soil layer, 34.4% tending thinning significantly increased the SOC storage (*p<* 0.05), while in the 10–20 cm soil layer and the total soil carbon storage, there were no significant differences (*p* > 0.05). The carbon storage in the soil layer of 0–10 cm ranges from 17.99-68.27 t/ha, with a large variation range of 50.28 t/ha. At 34.4% thinning intensity, the soil carbon storage was the largest, more than twice that of the CK group. However, the carbon storage in both the low-intensity and high-intensity thinning groups was lower than that of the CK group to varying degrees. In the 10–20 cm soil layer, the carbon storage ranged from 11.01 to 20.12 t/a, with a smaller range of 9.11 t/a. However, the carbon storage in the five thinning groups was greater than that in the CK group to varying degrees. Total soil storage showed a trend similar to that of the surface soil (0–10 cm). Among the five thinning groups, only 34.4% thinning group had total carbon storage(83.80 t/ha) more than twice that of the CK group (43.99 t/ha) (*p<* 0.05), while the carbon storage in the other four thinning groups was lower than that of the CK group (*p* > 0.05).

**Table 11:** SOC storage at different thinning intensities.

Thinning intensity	Soil carbon storage (t/a)		Soil total carbon storage (t/a)
	0~10 cm	10~20 cm	
CK	32.97 ± 2.98 a*	11.01 ± 3.62 a	43.99 ± 4.12 a
16.7%	23.72 ± 8.5 a	13.66 ± 4.10 a	37.37 ± 11.38 a
25.5%	18.48 ± 10.60 a	20.12 ± 7.49 a	38.60 ± 8.03 a
34.4%	68.27 ± 11.28 b	15.53 ± 5.25 a	83.80 ± 16.47 b
49.6%	17.99 ± 7.44 a	14.01 ± 4.82 a	32.00 ± 12.24 a
59.9%	26.64 ± 7.27 a*	14.29 ± 4.92 a	40.94 ± 8.19 a

Different letters in the same column indicate significant differences among different thinning groups at the same soil layer (*p* < 0.05), while the same letter indicates no significant differences (*p* > 0.05); * indicates significant differences between different soil layers within the same thinning group (*p* < 0.05).

**Figure 6:**
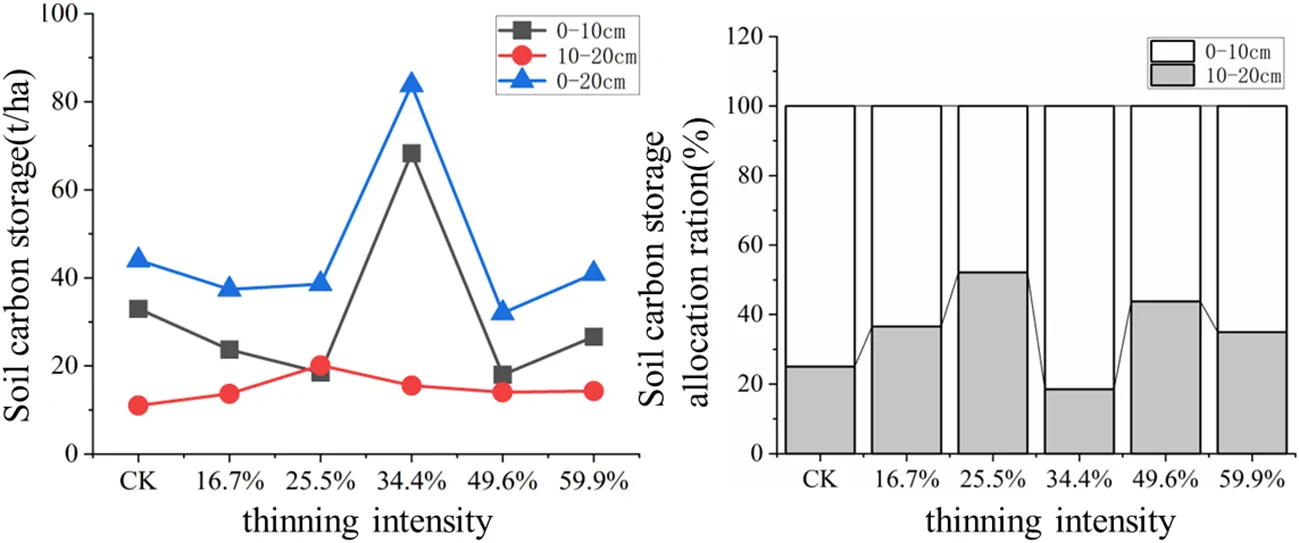
Soil carbon storage under different thinning intensities.

It can also be seen from **Table 11** that, except for the 25.5% thinning group, the spatial distribution of carbon storage in each layer in the other four thinning groups and the CK group was: 0–10 cm > 10–20 cm. In **Figure 6**, the variations of carbon storage in different soil layers under different thinning intensities and their spatial distribution patterns are visualized.

It can also be seen from **Figure 6** that the carbon storage in each layer differed. The carbon storage in the surface soil (0–10 cm) was generally greater than that in the 10–20 cm soil layer, and the carbon storage in the 0–10 cm soil layer changed in a “W” shape with the increasing thinning intensity. However, the carbon storage in the 10–20 cm soil showed an inverted “V” shape with increasing thinning intensity, characterized by an initial increase followed by a decrease, but the change trend was relatively slow and the amplitude is small. The variation trend of total carbon storage in the 0–20 cm soil with thinning intensity was the same as that of the 0–10 cm surface soil. However, at low thinning intensities, the variation trend was slower.

It can be seen from the spatial distribution ratio of soil carbon storage in **Figure 6** that, except for the 34.4% intensity thinning group, the proportion of soil carbon storage in the 0–10 cm layer in the other thinning groups was smaller than that in the CK group. Under different thinning intensities, the distribution ratio of carbon storage in each soil layer differed. With varying thinning intensity, the spatial allocation ratio of soil carbon storage in the 0–10 cm layer ranged from 48% to 81%. The variation pattern with increasing thinning intensity was the same as that of soil carbon storage in the 0–10 cm layer. This also demonstrates that in the soil carbon storage, the carbon storage in the surface soil (0–10 cm) plays a dominant role in the entire soil layer.

### 3.4 Correlation analysis between soil physical and chemical properties and soil carbon storage

The bivariate analysis results of the comprehensive physical and chemical properties and carbon storage of the soil in the tending thinning intensity sample plots are shown in **Table 12**. **Table 12** shows that the total soil carbon storage was extremely significantly positively correlated with soil moisture content and organic carbon content (*p<* 0.01), and significantly positively correlated with pH (*p<* 0.05). The soil carbon storage in the 0–10 cm layer was extremely significantly positively correlated with soil moisture content and SOC (*p<* 0.01). The carbon storage in the 10–20 cm soil was extremely significantly positively correlated with the maximum water-holding capacity and total P (*p<* 0.01), and significantly positively correlated with total K (*p<* 0.05).

**Table 12:** Correlation analysis between soil physical and chemical properties and soil carbon storage.

Variable	IF1	IF2	IF3	IF4	IF5	IF6	IF7	IF8	IF9	IF10	IF11	IF12	IF13	IF14	IF15	IF16	IF17
IF1	1																
IF2	-0.541	1															
IF3	-0.201	0.613	1														
IF4	0.178	0.638	0.634	1													
IF5	0.173	0.646	0.652	0.999**	1												
IF6	0.006	0.737	0.661	0.981**	0.977**	1											
IF7	0.79	-0.022	0.047	0.413	0.424	0.26	1										
IF8	0.987**	-0.63	-0.232	0.126	0.119	-0.046	0.694	1									
IF9	0.41	0.414	0.272	0.629	0.651	0.523	0.848*	0.303	1								
IF10	0.062	0.455	0.866*	0.614	0.647	0.559	0.363	0.026	0.588	1							
IF11	0.103	0.744	0.717	0.879*	0.896*	0.847*	0.562	0.005	0.827*	0.792	1						
IF12	0.401	0.384	0.759	0.885*	0.891*	0.818*	0.535	0.359	0.59	0.782	0.828*	1					
IF13	0.455	0.181	0.69	0.49	0.514	0.386	0.68	0.386	0.629	0.837*	0.701	0.792	1				
IF14	0.15	-0.146	0.609	0.146	0.174	0.07	0.055	0.21	0.079	0.752	0.217	0.482	0.62	1			
IF15	0.998**	-0.536	-0.2	0.198	0.19	0.032	0.768	0.988**	0.381	0.038	0.096	0.412	0.433	0.128	1		
IF16	-0.07	0.698	0.858*	0.698	0.728	0.675	0.388	-0.145	0.693	0.943**	0.915*	0.751	0.773	0.498	-0.091	1	
IF17	0.989**	-0.426	-0.063	0.31	0.306	0.14	0.831*	0.966**	0.493	0.189	0.242	0.532	0.557	0.207	0.987**	0.068	1

Bold values indicate indicators that are significantly correlated with carbon storage metrics. * indicates significant correlation (*p* < 0.05), and ** indicates extremely significant correlation (*p* < 0.01).) F1: Moisture content; F2: Soil bulk density; F3: Maximum water holding capacity; F4: Total porosity F5: Capillary porosity; F6: Non-capillary porosity; F7: pH value; F8: Organic matter; F9: total N; F10: total P; F11: total K; F12: Available N; F13: Available P; F14: Available K; F15:0-10 cm soil carbon storage; F16:10-20 cm soil carbon storage; F17: Total carbon storage in 0-20 cm soil.

Since the experiment integrated all the data from the tending thinning intensity plots, the correlation was not related to the thinning intensity. For plots with different thinning intensities, the correlation between soil physical and chemical properties and carbon storage was consistent. The results indicated that tending thinning changed soil moisture content, maximum water-holding capacity, organic matter content, pH, total P and total K nutrient contents, and thereby altered soil carbon storage, with a relatively small relationship with available soil nutrients. It should be noted that some correlations, particularly between SOC and carbon storage, may partly reflect mathematical coupling, as carbon storage calculations incorporate SOC values. Therefore, these correlations should be interpreted as confirming internal consistency of measurements rather than independent ecological relationships.

## 4 Discussion

This study systematically investigates the effects of tending thinning on the soil carbon cycle in cold-temperate natural secondary forests through an integrated multidisciplinary approach. Our findings not only confirm the nonlinear relationship between thinning intensity and soil carbon storage but, more importantly, identify 34.4% as the thinning intensity that maximized soil carbon storage under the conditions of this study, and elucidate the underlying “water-carbon coupling” mechanism. Furthermore, we have innovatively combined near-infrared spectroscopy with artificial intelligence algorithms to develop a high-precision predictive model for SOC content, providing technical support for accurate forest carbon sink monitoring.

### 4.1 Nonlinear effects of thinning intensity and optimal threshold

Our research reveals that thinning effects on soil carbon storage follow a distinct nonlinear pattern with a clear optimal range where carbon storage is maximized (Li et al., 2025). Specifically, only the 34.4% thinning intensity significantly enhanced carbon storage in both the 0–10 cm and 0–20 cm soil layers, while other intensities resulted in lower or statistically insignificant changes compared to the control. This finding provides crucial empirical evidence for resolving international debates regarding thinning effects (Yan et al., 2025). The variation in carbon storage across different thinning intensities primarily stems from their differential impacts on micro-environmental conditions (Lv et al., 2025). Moderate thinning (34.4%) creates a carbon-favorable microenvironment through optimized stand structure: appropriate canopy opening improves light availability, stimulating understory vegetation growth and litter input, while avoiding the negative consequences of excessive thinning such as soil temperature fluctuations, enhanced evaporation, and potential erosion (Juyang et al., 2025). This “moderate disturbance” state represents the key to maximizing carbon accumulation. Our results partially align with *Federico Romeo and Giovanna Settineri* et al (Romeo et al., 2020), who reported increased soil carbon storage under high-intensity thinning (45%), but simultaneously demonstrate that not all high-intensity treatments are effective, as evidenced by the 59.9% intensity failing to promote carbon accumulation. This suggests the existence of an ecological threshold (Li et al., 2025). Insufficient thinning (e.g., 16.7%) may inadequately improve light and microclimatic conditions, thereby limiting understory development and litter input (Juyang et al., 2025). Conversely, excessive thinning (e.g., 59.9%) can cause substantial disturbance leading to soil temperature instability, accelerated organic matter decomposition, and potential erosion, ultimately offsetting any positive effects from increased litter input (Lan et al., 2024). Therefore, our study emphasizes the critical importance of precisely quantifying and implementing appropriate thinning intensities in forest management strategies, as both excessively high and low intensities may fail to enhance carbon sequestration (Yan et al., 2025; Zaher et al., 2025). It is important to note that our conclusion regarding the 34.4% intensity is specific to the conditions of this study (stand type, climate zone, and soil conditions) and may not be directly generalizable to other forest types without further validation.

### 4.2 Multi-scale drivers of soil carbon accumulation

Correlation analysis revealed potential drivers governing soil carbon storage dynamics. In surface soils (0–10 cm), carbon storage is primarily associated with moisture and organic matter content, suggesting the potential dominance of water-carbon coupling (Acharya et al., 2025). This clearly explains why the 34.4% thinning treatment showed superior performance, as it maintained the highest soil moisture content (56.67%) among all treatments. Moisture serves as a key limiting factor for microbial activity and organic matter decomposition (Wen et al., 2025). Moderate moisture enhancement promotes litter decomposition and humification processes, potentially converting thinning-generated litter (e.g., branches and leaves) into stable SOC (Wang et al., 2025a). This hypothesized mechanism aligns with findings from numerous previous studies, collectively suggesting the central role of moisture in forest soil carbon cycling (Xue et al., 2022; Lin et al., 2024b; Qubaja et al., 2025). Furthermore, carbon storage in deeper soil layers (10–20 cm) showed significant correlations with maximum water-holding capacity, total phosphorus, and total potassium, indicating that carbon stabilization in subsurface layers may be more associated with soil structure and nutrient status (Tang et al., 2025; Yang et al., 2025). Enhanced root turnover post-thinning and increased leaching of dissolved organic carbon likely represent important sources for deep soil carbon pools, while sequestration may be regulated by soil physical structure and nutrient availability (Pei et al., 2025; Zhang et al., 2025). However, it must be emphasized that correlation analysis alone cannot establish causality. The observed associations between soil properties and carbon storage are consistent with, but do not prove, the hypothesized mechanistic pathways. Future studies incorporating direct measurements of microbial community structure, enzyme activities, and root dynamics are needed to validate these proposed mechanisms.

### 4.3 Interpretations of soil carbon distribution patterns

An intriguing finding of this study is the observation that under 34.4% thinning, surface soil carbon (0–10 cm) increased substantially while subsurface carbon (10–20 cm) was lower than in the control and some low-intensity treatments. This pattern, which appears to contradict typical vertical distribution of SOC, may be explained by several factors. First, the substantial increase in surface litter input under 34.4% thinning likely concentrated carbon accumulation in the topsoil layer before significant downward transport occurred. Second, differences in soil moisture regimes among treatments may have affected vertical water movement and dissolved organic carbon transport. Third, root distribution patterns likely differed among treatments, with potential differences in root turnover contributing to carbon inputs at different depths. Fourth, enhanced aeration and microbial activity in the subsoil under 34.4% thinning may have accelerated decomposition of existing subsoil carbon, offsetting some of the inputs from aboveground sources. This interpretation, while plausible, requires direct measurement of root biomass, microbial activity, and carbon fraction dynamics to confirm. These observations highlight the complexity of carbon dynamics across soil profiles and the need for depth-specific investigations in future thinning studies.

### 4.4 Innovations, applications and generalization considerations of the NIRS and BPNN model in forest carbon monitoring

A significant innovation of this study lies in integrating thinning experiments with advanced near-infrared spectroscopy to develop a high-precision soil carbon prediction model (Wan et al., 2024). The BPNN model with OSC preprocessing achieved a prediction set correlation coefficient of 0.9293, outperforming numerous comparable studies (Dotto et al., 2018; Nawar and Mouazen, 2019; Seema et al., 2020). The BPNN demonstrated superior capability in handling nonlinear spectral relationships, enabling capture of complex spectral features associated with carbon content (Mansour et al., 2023; Torresani et al., 2023). This breakthrough manifests at three levels: methodologically, OSC preprocessing effectively eliminates light scattering effects from soil particle heterogeneity, while the BP network’s powerful nonlinear fitting capability enables accurate mapping between spectral data and carbon content (Wan et al., 2024); technically, we established a complete workflow from spectral acquisition to carbon prediction, laying foundation for standardized applications (Li et al., 2024); practically, the method reduces detection time from hours to minutes per sample while eliminating chemical reagents, aligning with green detection trends (Zhang et al., 2024a). With advancing IoT and mobile technologies, this approach shows potential for integration with drone remote sensing and ground platforms to form an integrated space-air-ground forest carbon monitoring network.

While the BPNN model developed in this study demonstrates good predictive performance on the independent validation set (*R* = 0.9293, RPD = 2.8), several factors should be considered when applying this model to new contexts. Regarding the potential overfitting concern with the high-dimensional input (2,151 wavelengths) and relatively limited sample size (48 training samples), we acknowledge that this configuration carries a theoretical risk of overfitting. However, we emphasize that early stopping was employed during training to prevent overtraining, with 20% of training data held out as an internal validation subset for monitoring generalization performance. More importantly, the model’s strong performance on the completely independent prediction set (*R* = 0.9293, RMSEP = 0.0064, RPD = 2.8) provides empirical evidence that the model generalizes well within the study system. The high collinearity among adjacent spectral wavelengths also means that the effective degrees of freedom are substantially lower than the nominal 2,151 variables, reducing the practical risk of overfitting. Nevertheless, future work with larger sample sizes would allow for more robust model validation and the potential application of more rigorous regularization techniques, such as dropout or L1/L2 regularization.

The model was trained and validated using samples from a single forest type (larch-birch secondary forests in the Greater Khingan Mountains) and a specific soil type (acidic mountainous brown coniferous forest soil). Its applicability to other climatic zones, forest stand types, and soil types requires further validation. Factors such as differences in soil mineral composition, organic matter quality, and moisture regimes can affect spectral signatures and potentially reduce prediction accuracy when the model is applied to new environments. In addition, the spectral preprocessing parameters and model architecture optimized for this specific dataset may not be optimal for other soil types, and recalibration may be necessary.

For practical application to new regions or forest types, we recommend conducting a preliminary validation using a small set of local samples with known carbon contents (e.g., 20–30 samples). If the prediction accuracy (as measured by RMSEP or RPD) falls below acceptable thresholds, model recalibration or transfer learning approaches should be considered. This pragmatic recommendation is intended to guide future applications of the NIRS-BPNN approach and to ensure that the model is used appropriately within its validated scope.

Furthermore, the current model was developed using laboratory-based spectral measurements under controlled conditions. Field-based applications would require additional considerations, including the effects of soil moisture, temperature, and surface conditions on spectral acquisition. The development of field-deployable NIRS systems with integrated calibration transfer methods represents a promising direction for future research.

### 4.5 Limitations and future perspectives

Despite meaningful findings, several limitations warrant attention. First, the 12-year study period may not fully capture long-term thinning effects, as ecological responses often exhibit temporal accumulation and hysteresis. Future research should establish long-term monitoring stations to track soil carbon pool dynamics post-thinning. Second, focusing on total SOC limits mechanistic understanding, as different organic carbon fractions (e.g., particulate organic carbon, mineral-associated organic carbon) may respond differently to management. Future work should integrate molecular and physical fractionation techniques to investigate how thinning affects organic carbon composition and stability. Third, while our NIRS model demonstrates high accuracy, its generalizability requires validation across broader geographical regions and forest types as discussed above. Future directions should include collecting samples from diverse climatic zones and soil types to build more universal and robust prediction models. Fourth, better integration of biological factors is needed, particularly systematic analysis of relationships between soil microbial community structure, enzyme activities, and carbon cycling processes. Direct measurements of litter input, root biomass, and microbial community composition would substantially strengthen mechanistic interpretations. Fifth, our study was conducted at a single site with specific environmental conditions. The observed patterns and the identification of 34.4% as the intensity associated with highest carbon storage are specific to this study context and may vary under different climatic, edaphic, or stand conditions. Finally, the mathematical coupling between SOC and carbon storage in correlation analyses should be carefully considered when interpreting relationships between soil properties and carbon storage.

Through multidisciplinary approaches, this study systematically reveals thinning effects on soil carbon cycling, develops novel monitoring technologies, and proposes innovative management implications. These achievements provide not only guidance for regional forest management but also important references for global forest carbon research and climate change mitigation. Future studies should build on this foundation by integrating multi-process and multi-scale observations to deepen understanding of forest carbon mechanisms and promote wider application of research outcomes.

## 5 Conclusion

This study demonstrates that under the conditions of this study, the 34.4% thinning intensity was associated with the highest soil carbon sequestration in the natural secondary larch-birch forests of the Greater Khingan Mountains. This management strategy significantly increased total soil carbon storage to 83.80 t/ha, with surface layer (0–10 cm) accumulation exceeding subsurface storage by more than fourfold.

The improved carbon sequestration under 34.4% thinning intensity was primarily associated with enhanced soil moisture, organic matter content, and nutrient availability. Correlation analysis confirmed that these parameters, along with pH, water-holding capacity, and phosphorus-potassium levels, served as potential determinants of carbon storage dynamics. However, it should be noted that correlation does not establish causation, and these relationships warrant further mechanistic investigation.

Furthermore, we developed an efficient monitoring approach through a near-infrared spectroscopy model integrated with backpropagation neural network (*Rp* = 0.9293, *R²p* = 0.8635, RMSEP = 0.0064, RPD = 2.8), which provides a reliable and rapid method for predicting SOC content in this forest type. This model offers potential for application in large-scale soil carbon monitoring, though validation across different forest types and soil conditions is recommended before widespread use.

These findings support the implementation of moderate-intensity thinning as a viable forest management practice for enhancing carbon sequestration in similar secondary forests, while the established prediction model offers an effective tool for soil carbon monitoring. However, the specific intensity that maximizes carbon storage may vary across different forest types, climate zones, and soil conditions, and should be determined through site-specific experimentation.

## Data Availability

The raw data supporting the conclusions of this article will be made available by the authors, without undue reservation.
